# Healthcare Needs, Experiences and Satisfaction after Terrorism: A Longitudinal Study of Survivors from the Utøya Attack

**DOI:** 10.3389/fpsyg.2016.01809

**Published:** 2016-11-24

**Authors:** Lise E. Stene, Tore Wentzel-Larsen, Grete Dyb

**Affiliations:** ^1^Norwegian Centre for Violence and Traumatic Stress StudiesOslo, Norway; ^2^Centre for Child and Adolescent Mental Health, Eastern and Southern NorwayOslo, Norway; ^3^Faculty of Medicine, Institute of Clinical Medicine, University of OsloOslo, Norway

**Keywords:** health services research, healthcare quality, mass casualty incidents, disaster planning, PTSD, psychosomatic medicine

## Abstract

**Background:** Public health outreach programs have been developed in order to ensure that needs are met after disasters. However, little is known about survivors' experiences with post-terror healthcare. In the present study, our objectives were to (1) describe survivors' experiences with post-terror healthcare, (2) identify factors associated with reports of unmet healthcare needs, and (3) examine the relationship between socio-demographic characteristics, healthcare experiences and satisfaction.

**Methods:** Our study comprised three waves of semi-structured interviews with 261/490 (53%) survivors of the Utøya mass shooting. We applied Pearson's chi-squared tests (categorical variables) and independent *t*-tests (continuous variables) to compare survivors by whether or not they reported higher perceived needs than received help for psychological reactions and physical health problems, respectively. Ordinal regression analyses were applied to examine whether socio-demographic characteristics and healthcare experiences were associated with dissatisfaction.

**Results:** Altogether 127 (49%) survivors reported very high/high help needs for psychological reactions, and 43 (17%) for attack-related physical health problems. Unmet healthcare needs were associated with higher levels of posttraumatic stress, psychological distress, somatic symptoms and less social support. Survivors with immigrant backgrounds and injured survivors who were not admitted to hospital reported unmet needs for physical health problems more often. After adjustments for socio-demographic characteristics, immigrant origin was associated with dissatisfaction with post-terror healthcare. After additionally adjusting for healthcare experiences, poor rating of the overall organization and accessibility of healthcare remained significantly associated with dissatisfaction.

**Conclusions:** Most survivors were satisfied with the post-terror healthcare they received, yet our findings indicate that increased attention to the physical health of non-hospitalized terrorism survivors is required. Furthermore, in future outreach, particular attention should be paid to the healthcare needs of survivors with immigrant background.

## Introduction

Experiencing a terrorist attack may have a severe effect on mental and physical health (North et al., 1999; Feng et al., 2006; Miguel-Tobal et al., 2006; Tucker et al., 2007; Whalley and Brewin, 2007; Neria et al., 2011; Dyb et al., 2014). Despite efforts to develop public health outreach programs to respond to survivors' healthcare requirements, (Felton, 2002; Brewin et al., 2010) unmet needs have been reported in the aftermath of terrorism (Pfefferbaum et al., 2003; Fairbrother et al., 2004). Although, evaluation of health services is necessary for their improvement, there has been little research on post-terror healthcare provision. Existing studies have primarily comprised cross-sectional assessments of posttraumatic stress reactions and use of counseling services. Health authorities increasingly recognize patients' perceived experiences and satisfaction as important aspects of healthcare quality (Donabedian, 1988). Satisfaction is a broad concept that may reflect multiple factors; including the quality of care and the patient's expectations and preferences (Bleich et al., 2009). It is therefore valuable to assess different types of experiences with the post-terror outreach that may be important from the survivors' perspective and may contribute to satisfaction with healthcare. A better understanding of survivors' experiences and satisfaction with post-terror outreach is essential for identifying the aspects of healthcare that may be important for meeting survivors' needs and strengthening public health preparedness for future attacks. This study investigates the healthcare needs, experiences and satisfaction of the Utøya attack survivors.

On July 22, 2011, a solitary right-wing terrorist perpetrated a shooting spree at the summer camp of a political youth organization on the Utøya islet. During the nearly 1.5 h long shooting, 69 were killed, many lost friends or family members and many were injured or risked drowning in attempts to swim to safety. The 495 survivors came from rural and urban municipalities throughout Norway. In order to anticipate the unmet needs observed after previous terrorist attacks, a primary care based proactive outreach program was created to identify survivors who would develop mental health problems and need specialized treatment. The Norwegian Directorate of Health recommended that municipal crisis teams contact all survivors directly after the attack, and that all survivors be appointed a municipal contact person. The contact person was to ensure continuity in the follow-up throughout the first year, and set up standardized screening assessments at 5–6 weeks, 3, and 12 months after the attack (Norwegian Directorate of Health, 2012). Prior research has shown that all survivors received some type of healthcare in the aftermath of the Utøya attack, though an important minority was not proactively followed-up as recommended (Dyb et al., 2011; Stene and Dyb, 2015). The survivors' perceived needs, experiences and satisfaction with post-terror healthcare provision have not yet been investigated. The aims of this study were to:

- Describe the survivors' perceived needs, experiences and satisfaction with post-terror healthcare.
- Identify factors associated with reporting unmet healthcare needs.
- Examine whether satisfaction with post-terror follow-up was related to socio-demographic factors and experiences with healthcare.

## Materials and methods

### Participants and procedures

Police records were used to identify 495 survivors who had been on the Utøya islet during the shooting. We excluded four survivors aged <13 years and one living abroad, hence study invitations were sent by mail to 490 survivors. Semi-structured face-to-face interviews were performed by trained clinicians at 4–5 months (wave 1), 14–15 months (wave 2), and 31–32 months (wave 3) after the attack. Questions about healthcare needs, experiences and satisfaction were included at wave 3. The study had an open cohort design in which all the eligible survivors (*n* = 490) were invited to participate at waves 1 and 2. At wave 3 invitations were sent to the 355 (72%) survivors who participated at wave 1 or 2. Altogether, 261 (53%) survivors participated at wave 3 and were included in this study. There were no significant differences between participating and non-participating survivors (*n* = 229) with respect to gender (52 vs. 57% male survivors, *p* = 0.258), age (mean 19.5 (SD 4.8) vs. 19.0 (3.9) years, *p* = 0.189), hospitalization after the attack (7 vs. 8%, *p* = 0.450) and peripheral residence (13 vs. 18%, *p* = 0.162). Survivors who were lost to follow-up after the first two study waves (*n* = 94) were significantly more likely to be of non-Norwegian origin than survivors in our sample (*p* = 0.002). However, they did not differ significantly in their levels of posttraumatic distress, anxiety/depression, somatic symptoms and social support (*p* ≥ 0.208). Participants aged ≥16 years provided written informed consent. Parental consent was required before survivors aged <16 years could participate in the study. The Regional Committees for Medical and Health Research Ethics South East and North in Norway approved the study. The study procedures have been described in detail in a previous study (Stene and Dyb, 2016).

### Variables

#### Healthcare needs, experiences, and satisfaction

There are no standardized methods for assessing healthcare experiences among survivors of mass-casualty incidents. Our questions on perceived experiences and satisfaction with follow-up were developed from questions included in national surveys of patient experiences in Norway designed by the Norwegian Knowledge Centre for the Health Services (Garratt et al., 2011). The wording of some questions was adjusted to fit the post-disaster setting. Questions on healthcare needs, experiences and satisfaction had a five-point response scale, ranging from “not at all” to “to a very large extent,” which has been shown to be satisfactory for assessing patient experiences (Garratt et al., 2011). The questions are given in Box 1. We did separate assessments for the perceived needs and receipt of help or treatment for attack-related psychological reactions and physical health problems. The survivors were first asked to rate the extent to which they had required help, and immediately afterwards they were asked to rate the extent to which they had received help. If the survivors' scores for required help were higher than the scores for help received, the response was categorized as unmet needs for psychological reactions and physical health problems.

Box 1 **Questions on healthcare needs, experiences and satisfaction**

***Healthcare needs***

I will now ask you some questions about your need for help. With respect to what you experienced at Utøya, have you…

- Felt that you needed help or treatment for psychological reactions/problems?
- Received help from healthcare practitioners for psychological reactions/problems?
- Felt that you needed help or treatment for bodily/physical health problems?
- Received help from healthcare practitioners for bodily/physical health problems?
- Do you currently feel that you need help or treatment for psychological reactions/problems?
- Do you currently feel that you need help or treatment for bodily/physical health problems?

***Response alternatives:***

Not at all/To a small extent/To some extent/To a large extent/To a very large extent

***Healthcare experiences***

We would like to know about your overall experience with the public help services in relation to what you went through at Utøya.

- Did you find the help services to be well organized?
- Were you offered help without having to ask for it?
- How satisfied are you with the accessibility of the help services?
- Were you treated with care and consideration?
- Were you involved in decisions regarding your treatment/follow-up?
- Did you get enough time to talk and interact with healthcare practitioners?
- Did a single representative from the help services have the main responsibility for your follow-up?

***Response alternatives:***

Not at all/To a small extent/To some extent/To a large extent/To a very large extent

***Satisfaction***

Overall, were the help and treatment you received after the terrorist attack satisfactory?

***Response alternatives:***

Not at all/To a small extent/To some extent/To a large extent/To a very large extent

***Other questions regarding post-terror follow-up***

Overall, what benefit have you had from the help you received from public services after the terrorist attack?

Response alternatives: No/Little/Some/Large/Very large benefit

Did you have to wait to be offered help from the public services after the terrorist attack?

Response alternatives: No/Yes, but not for long/Yes, quite long/Yes, far too long

#### Psychological assessment

Posttraumatic stress reactions in the past month were measured using the University of California at Los Angeles Post-traumatic Stress Disorder (PTSD) Reaction Index (UCLA PTSD-RI) (Steinberg et al., 2004). The total score covers 17 items that conform to the DSM–IV symptoms of PTSD rated on a five-point Likert scale ranging from 0 (never) to 4 (most of the time) (American Psychiatric Association, 2000). Three items have two alternative wordings that are assessed by the item with the highest score. It has been documented that a mean score of 38 or greater on the PTSD-RI has the greatest sensitivity and specificity for detecting PTSD (Steinberg et al., 2004). The mean score was used in the analyses (Cronbach's alphas: waves 1 and 2 = 0.89 and wave 3 = 0.91). Symptoms of anxiety and depression were assessed with the Hopkins Symptom Checklist-8 (SCL-8). This is a short version of the SCL-25 which measures symptoms of depression/anxiety in the past 2 weeks using eight items scored from 1 (not bothered) to 4 (very bothered) (Derogatis et al., 1974). The mean score of five of the Hopkins Symptom Checklist items can be dichotomized by a validated cut-off at >2.0 to serve as a measure of anxiety and depression (Tambs and Moum, 1993). We applied the mean score in the analyses (Cronbach's alphas: wave 1 = 0.85, wave 2 = 0.89 and wave 3 = 0.90). The short versions of the Hopkins Symptom Checklist have shown high psychometric qualities in population-based studies (Strand et al., 2003). Somatic symptoms in the past 2 weeks were measured using a short version of the Children's Somatic Symptoms Inventory (CSSI-8) (Walker et al., 2008). The eight items assessed pain in the stomach, head, lower back, and arms/legs; faintness/dizziness; rapid heartbeat; nausea/stomach problems; and weakness. The items were scored on a scale from 1 (not bothered) to 4 (very bothered). We used the mean score in the analyses (Cronbach's alphas: wave 1 = 0.77, wave 2 = 0.78 and wave 3 = 0.77). Social support was assessed using seven items from the Duke University of North Carolina Functional Social Support Questionnaire (FSSQ-7) scored from 1 (much less than I would like) to 5 (as much as I would like)(Broadhead et al., 1988) (Cronbach's alphas: wave 1 = 0.79, wave 2 = 0.77 and wave 3 = 0.80).

#### Other characteristics

We obtained information on age, gender and place of residence from police records. The other data were collected during the first interview. Age was assessed at the time of the attack as a continuous variable with one decimal in the analyses. If the survivor's home municipality was located more than 45 min traveling time from a settlement with at least 15000 inhabitants, it was defined as peripheral residence according to Statistics Norway's classification of centrality (Statistics Norway Centrality, 2008). Survivors with both parents born abroad were classified as having non-Norwegian origin. Financial status was assessed by asking survivors how they perceived their own (survivors who did not live with parents) or their parents' (survivors who lived with parents) economic status compared to others. The five response categories were dichotomized into financially disadvantaged (much or somewhat poorer) or not (similar, somewhat better, and much better). Physical injury comprised three categories. Survivors who were hospitalized directly after the attack because of physical injuries were classified as hospitalized. Hospitalization was verified by hospital records. Survivors who were not hospitalized and answered yes to the question “Were you physically injured and required medical assistance?” were classified as injured but not hospitalized, and survivors who answered no were classified as not injured.

### Data analysis

We did descriptive analyses of the survivors' perceived healthcare needs, experiences and satisfaction related to the post-disaster follow-up. Pearson's chi-squared tests (categorical variables) and independent *t*-tests (continuous variables) were used to compare survivors according to whether or not they reported higher perceived needs than received help for psychological reactions and physical health problems, respectively. Furthermore, we used unadjusted and adjusted ordinal regression analyses to examine whether socio-demographic factors and perceived experiences were related to levels of dissatisfaction. We applied two multivariable regression models. Model 1 adjusted for socio-demographic variables, including age, gender, origin, peripheral residence and self-perceived economic status. Model 2 adjusted for the survivors' healthcare experiences in addition to all variables in Model 1. Due to sample size constraints, questions on healthcare experiences were reduced from five to three categories, where “not at all” and “to a small extent” were combined as well as “to a large extent” and “to a very large extent.” A hierarchical cluster analysis using Spearman correlation of the questions on healthcare experiences identified a homogenous clustering for the questions about satisfaction with the accessibility of services and being given enough time to talk and interact with healthcare practitioners. Consequently, we applied the mean score of these items as a continuous variable in the analyses. The percentages and analyses were based on the total number of answers for each item. Due to non-participation, we lacked data on symptoms and social support for 20 (7.7%) survivors at wave 1 and 34 (13.0%) survivors at wave 2. There was little missing data among those who participated in waves 1 and 2, and no respondents had more than two items with missing data in any of the above-mentioned scales (PTSD-RI, SCL-8, CSSI-8 and FSSQ-7). If a respondent had missing data on one or two items, the mean scores of the answered items were used in the analyses. We presented the crude and adjusted odds ratios (OR) with 95% confidence intervals (CI). The analyses were performed with IBM SPSS version 22 and R version 3.3.3 (The R Foundation for Statistical Computing, Vienna, Austria).

## Results

**Figure 1:**
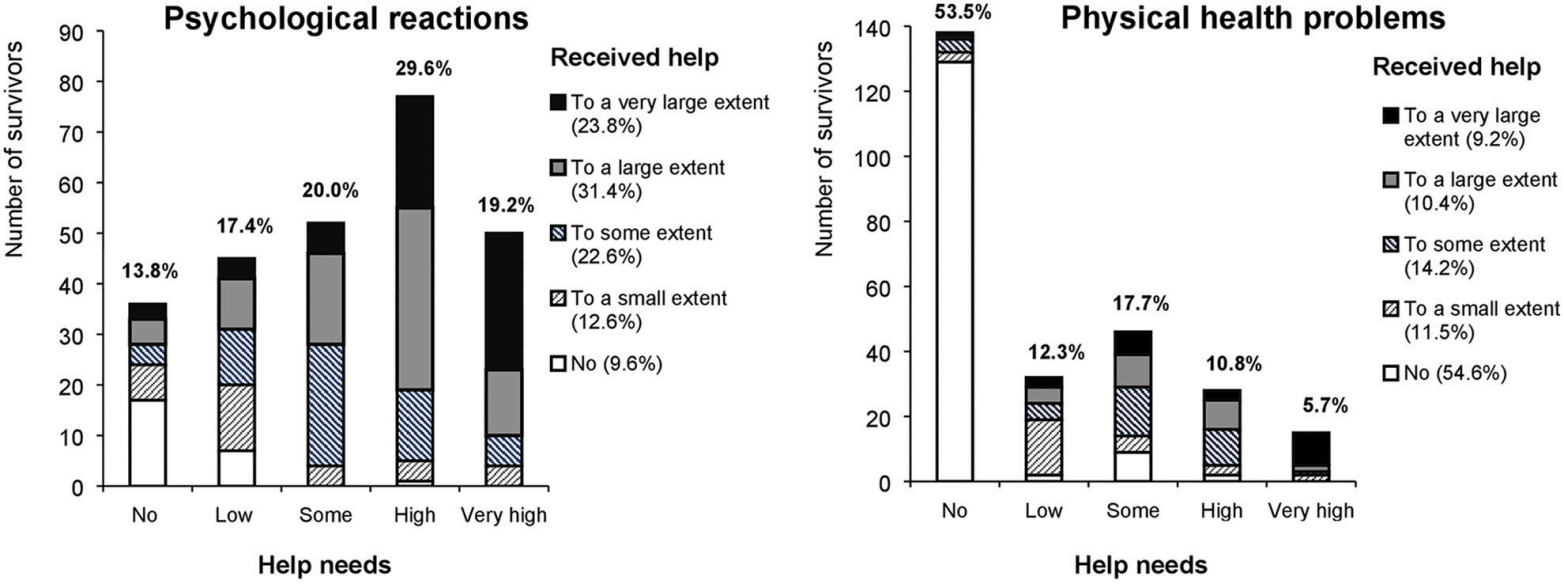
**The survivors' self-perceived overall needs, and received help/treatment, for psychological reactions (***n*** = 260) and physical health problems (***n*** = 259) related to the Utøya attack**.

Characteristics of our sample are presented in Table 1. Two and a half years after the attack, 125 (48.4%) survivors rated their health as excellent or very good. However, 98 (37.7%) survivors reported that their health had worsened after the attack. Furthermore, 43 (16.5%) survivors reported very high/high current help needs for psychological reactions, and, 24 (9.2%) reported very high/high current help needs for physical health problems. In all, 22 (8.6%) survivors reported that they had to wait too long or quite long before they were offered help. Altogether, 133 (51.6%) survivors reported very large/large benefit, whereas 45 (17.4%) reported little or no benefit from the overall help they had received after the attack. The number of survivors with symptoms above the clinical cut-off for PTSD at the respective survey waves were 53/241 (22.0%) at wave 1, 19/227 (8.4%) at wave 2, and 20/261 (7.7%) at wave 3. Furthermore, the number of survivors with clinical levels of anxiety and depression symptoms were 108/241 (44.8%) at wave 1, 66/227 (29.1%) at wave 2, and 64/261 (24.5%) at wave 3. Figure 1 presents the survivors' self-perceived overall attack-related needs and received help/treatment. With respect to psychological reactions, 53 (20.4%) survivors scored their healthcare needs as greater, and 90 (34.6%) as less, than the help they had received. As for attack-related physical health problems, 37 (14.3%) survivors rated their healthcare needs as greater, and 42 (16.2%) as less, than what they had received. Overall, 13 (5.0%) survivors scored their healthcare needs as greater than the help they had received for both psychological reactions and physical health problems. Altogether, 21 (48.8%) of the 43 survivors who reported very high/high help needs due to attack-related physical health problems had been physically injured during the attack.

**Table 1:** **Characteristics of our study sample of 261 (53%) survivors from the Utøya attack**.

**Characteristics of survivors**		***n***	**(%)**
Male gender (*n* = 261)		136	(52.1)
Age at attack (*n* = 261)	<18 years	116	(44.4)
	18–25 years	126	(48.3)
	≥26 years	19	(7.3)
Non-Norwegian origin (*n* = 257)		22	(8.6)
Financially disadvantaged (*n* = 255)		51	(20.0)
Peripheral residence (*n* = 259)		34	(13.1)
Self-perceived health (*n* = 258)	Excellent	28	(10.9)
	Very good	97	(37.6)
	Good	88	(34.1)
	Somewhat good	34	(13.2)
	Poor	11	(4.3)
Current health compared to	Much better	15	(5.8)
before attack (*n* = 260)	A little better	37	(14.2)
	Similar	110	(42.3)
	A little worse	76	(29.2)
	Much worse	22	(8.5)
Perceived help needs for psychological	Very high	18	(6.9)
reactions after 2.5 years (*n* = 261)	High	25	(9.6)
	Some	35	(13.4)
	Low	66	(25.3)
	No	117	(44.8)
Perceived help needs for physical health	Very high	7	(2.7)
problems after 2.5 years (*n* = 261)	High	17	(6.5)
	Some	32	(12.3)
	Low	28	(10.7)
	No	177	(67.8)
Had to wait to be offered help (*n* = 254)	No	189	(74.4)
	Yes, but not for long	43	(16.9)
	Yes, for quite long	14	(5.5)
	Yes, for too long	8	(3.1)
Perceived benefit of help (*n* = 258)	No benefit	8	(3.1)
	Little	37	(14.3)
	Some	80	(31.0)
	Much	75	(29.1)
	Very much	58	(22.5)
Overall satisfaction (*n* = 260)	Very high	78	(30.0)
	High	97	(37.3)
	Moderate	44	(16.9)
	Small	24	(9.2)
	No	17	(6.5)

*Numbers may not add up to exactly 100% due to rounding errors*.

Table 2 displays characteristics of survivors who reported healthcare needs greater than the help they had received for attack-related psychological reactions and physical health problems. Survivors who reported unmet needs for psychological reactions were significantly more likely to report higher levels of posttraumatic stress and anxiety/depression both shortly after the attack and at wave 3, i.e., after approximately 2.5 years, than those who did not. They were also more likely to report higher levels of somatic symptoms, less social support and poorer general health at wave 3 than the other survivors. With respect to attack-related physical health problems, unmet needs were associated with higher levels of posttraumatic stress, anxiety/depression and somatic symptoms, and less social support at all survey waves. Unmet needs were also associated with non-Norwegian origin and poorer general health. Furthermore, there was a significant relationship between physical injuries and unmet needs. None of the survivors who were hospitalized due to injuries reported unmet needs for physical health problems, compared to 11 (36.7%) of those who were injured but not admitted to hospital and 26 (12.4%) of the survivors who were not injured.

**Table 2:** **Survivor characteristics by self-perceived unmet healthcare needs for attack-related psychological and physical health problems**.

		**Perceived needs higher than received help**					
		**Psychological reactions (*****n*** = **260)**			**Physical problems (*****n*** = **259)**		
**Characteristics**		**Yes, *n* = 53 (%/SD)**	**No, *n* = 207 (%/SD)**	***p*-value**	**Yes, *n* = 37 (%/SD)**	**No, *n* = 222 (%/SD)**	***p*-value**
Mean age at attack (*n* = 260)		20.0 (5.6)	19.3 (4.5)	0.380	18.5 (3.0)	19.6 (5.0)	0.163
Gender (*n* = 260)	Female	29 (54.7)	96 (46.4)	0.278	21 (56.8)	103 (46.4)	0.243
	Male	24 (45.3)	111 (53.6)		16 (43.2)	119 (53.6)	
Non-Norwegian origin (*n* = 256)	Yes	6 (11.8)	16 (7.8)	0.402	7 (18.9)	15 (6.9)	0.025
	No	45 (88.2)	189 (92.2)		30 (81.1)	203 (93.1)	
Financially disadvantaged (*n* = 254)	Yes	13 (24.5)	38 (18.9)	0.363	9 (24.3)	41 (19.0)	0.451
	No	40 (75.5)	163 (81.1)		28 (75.7)	175 (81.0)	
Peripheral residence (*n* = 258)	Yes	4 (7.7)	30 (14.6)	0.191	6 (16.7)	28 (12.7)	0.512
	No	48 (92.3)	176 (85.4)		30 (83.3)	193 (87.3)	
Injured (*n* = 260)	Yes, hospitalized	1 (1.9)	16 (7.7)	0.332	0 (0.0)	17 (7.7)	0.001
	Yes, not hospitalized	6 (11.3)	23 (11.1)		11 (29.7)	18 (8.1)	
	Not injured	46 (86.8)	168 (81.2)		26 (70.3)	187 (84.2)	
General health (*n* = 258)	Very good/excellent	17 (32.7)	108 (52.7)	0.006	7 (18.9)	116 (53.0)	<0.001
	Good	19 (36.5)	68 (33.2)		16 (43.2)	72 (32.9)	
	Somewhat good/poor	16 (30.8)	29 (14.1)		14 (37.8)	31 (14.2)	
Health after attack (*n* = 260)	Poorer	29 (54.7)	69 (33.5)	0.017	24 (64.9)	74 (33.5)	0.001
	Same	17 (32.1)	92 (44.7)		10 (27.0)	98 (44.3)	
	Better	7 (13.2)	45 (21.8)		3 (8.1)	49 (22.2)	
PTSR mean score (PTSD-RI)	Wave 1 (*n* = 241)	30.8 (10.9)	25.1 (12.1)	0.003	32.3 (12.0)	25.1 (11.8)	0.001
	Wave 2 (*n* = 227)	24.1 (11.4)	20.0 (11.5)	0.030	28.1 (10.3)	19.7 (11.4)	<0.001
	Wave 3 (*n* = 261)	25.4 (13.8)	17.9 (11.6)	<0.001	28.7 (13.8)	17.9 (11.5)	<0.001
Anxiety/depression	Wave 1 (*n* = 241)	2.27 (0.68)	2.02 (0.64)	0.017	2.38 (0.61)	2.01 (0.64)	0.001
mean score (SCL-8)	Wave 2 (*n* = 227)	1.94 (0.72)	1.78 (0.63)	0.139	2.16 (0.62)	1.76 (0.64)	0.001
	Wave 3 (*n* = 261)	2.06 (0.77)	1.64 (0.60)	0.001	2.11 (0.67)	1.66 (0.63)	<0.001
Somatic symptoms	Wave 1 (*n* = 241)	1.81 (0.54)	1.70 (0.55)	0.221	2.02 (0.65)	1.67 (0.51)	<0.001
mean score (CSSI-8)	Wave 2 (*n* = 227)	1.68 (0.43)	1.64 (0.54)	0.653	1.89 (0.50)	1.61 (0.51)	0.006
	Wave 3 (*n* = 261)	1.67 (0.42)	1.51 (0.48)	0.026	1.85 (0.54)	1.49 (0.45)	<0.001
Social support	Wave 1 (*n* = 241)	4.45 (0.64)	4.60 (0.51)	0.125	4.26 (0.77)	4.62 (0.48)	0.010
mean score (FSSQ-7)	Wave 2 (*n* = 227)	4.50 (0.59)	4.62 (0.49)	0.179	4.35 (0.58)	4.63 (0.50)	0.007
	Wave 3 (*n* = 261)	4.25 (0.66)	4.61 (0.52)	<0.001	4.11 (0.82)	4.60 (0.49)	0.001

**Figure 2:**
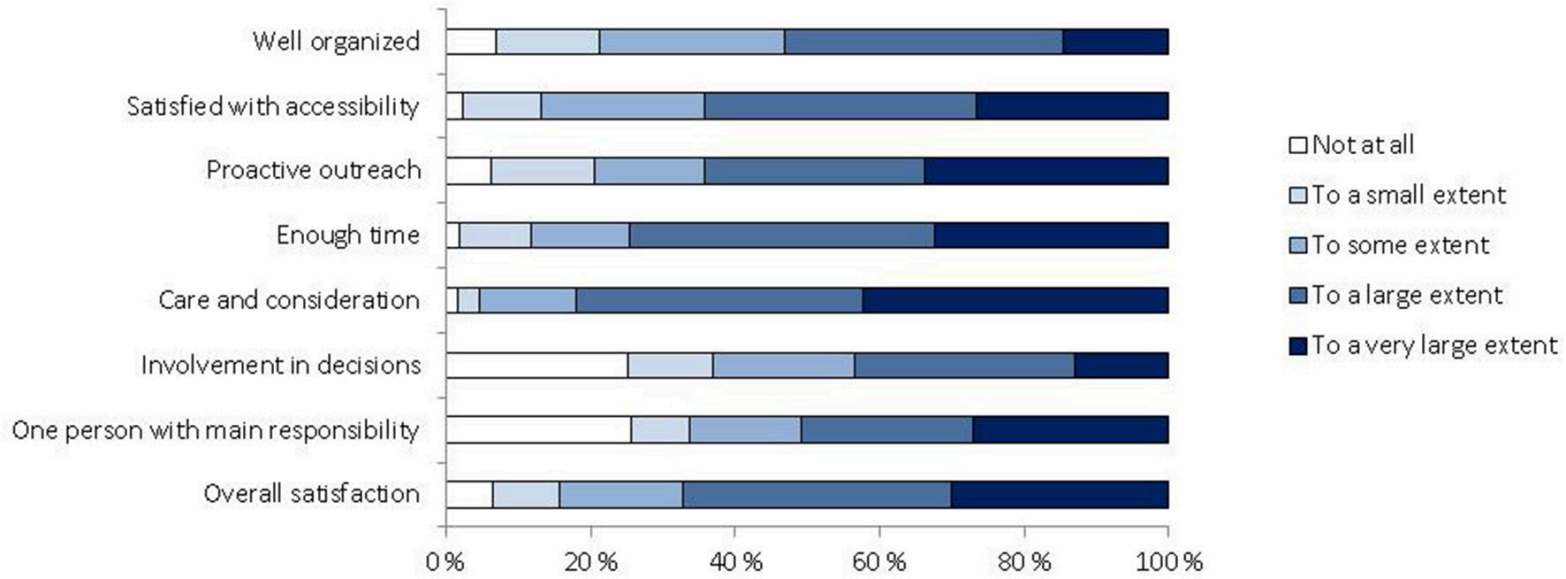
**Survivors' experiences and satisfaction with help services after the Utøya attack reported 31–32 months after the attack**.

Figure 2 displays the survivors' perceived experiences and overall satisfaction with the help services they received in relation to the Utøya attack. Overall, 78 (30.0%) survivors were satisfied to a very large extent, 97 (37.3%) to a large extent, 44 (16.9%) to some extent, 24 (9.2%) to a small extent and 17 (6.5 %) were not at all satisfied with the help services. No statistically significant relationship was found between satisfaction and self-perceived health or current health compared to before the attack (*p* = 0.267 and *p* = 0.444, respectively).

In the unadjusted ordinal regression analyses, non-Norwegian origin and low scores on all perceived experiences with healthcare were associated with overall dissatisfaction with help received after the attack (Table 3). In the multivariable ordinal regression model adjusting for socio-demographic factors (Model 1), non-Norwegian origin remained significantly associated with dissatisfaction. After additionally adjusting for perceived healthcare experiences (Model 2), only low scores on the overall organization and accessibility of healthcare remained significantly associated with dissatisfaction.

**Table 3:** **Multivariable ordinal regression models for dissatisfaction with help and treatment after the Utøya attack among 261 survivors**.

		**Unadjusted**			**Model 1 (*****n*** = **248)**			**Model 2 (*****n*** = **238)**		
		**OR**	**95% CI**	***p*-value**	**OR**	**95% CI**	***p*-value**	**OR**	**95% CI**	***p*-value**
**SOCIODEMOGRAPHIC CHARACTERISTICS**										
Age		1.006	(0.963–1.050)	0.788	1.061	(0.827–1.363)	0.641	1.077	(0.895–1.296)	0.435
Female gender		1.334	(0.857–2.075)	0.202	1.506	(0.945–2.400)	0.085	1.397	(0.812–2.403)	0.227
Non-Norwegian origin		2.423	(1.081–5.433)	0.032	2.813	(1.168–6.778)	0.021	2.379	(0.936–6.047)	0.069
Financially disadvantaged		1.737	(0.969–3.112)	0.064	1.750	(0.954–3.210)	0.071	1.396	(0.705–2.767)	0.339
Peripheral residence		1.111	(0.590–2.094)	0.744	1.204	(0.618–2.344)	0.586	1.179	(0.540–2.574)	0.679
**EXPERIENCES WITH HEALTH SERVICES**										
Well organized	Some vs. little extent	0.097	(0.046–0.205)	<0.001				0.164	(0.068–0.395)	<0.001
	High vs. little extent	0.024	(0.011–0.050)	<0.001				0.123	(0.047–0.321)	<0.001
Satisfied with accessibility/enough time		0.125	(0.088–0.178)	<0.001				0.220	(0.138–0.351)	<0.001
Proactive outreach	Some vs. little extent	0.545	(0.259–1.145)	0.111				1.048	(0.427–2.571)	0.919
	High vs. little extent	0.109	(0.058–0.206)	<0.001				0.688	(0.311–1.521)	0.355
Care and consideration	Some vs. little extent	0.466	(0.149–1.459)	0.190				1.804	(0.450–7.223)	0.405
	High vs. little extent	0.082	(0.029–0.232)	<0.001				3.122	(0.830–11.740)	0.092
Involvement in decisions	Some vs. little extent	0.533	(0.284–1.000)	0.050				0.820	(0.399–1.683)	0.588
	High vs. little extent	0.189	(0.110–0.325)	<0.001				0.538	(0.282–1.027)	0.060
One person with main responsibility	Some vs. little extent	0.421	(0.215–0.824)	0.012				1.080	(0.487–2.395)	0.851
	High vs. little extent	0.116	(0.066–0.204)	<0.001				0.507	(0.257–1.001)	0.050

*Model 1 adjusted for sociodemographic characteristics. Model 2 adjusted for sociodemographic characteristics and experiences with health services. Overall statistical*.

significance for each experience with health services: All unadjusted p-values were <0.001. Model 2: Well organized: p < 0.001; Satisfied with accessibility/enough time: p < 0.001;

*Proactive outreach p = 0.488; Care and consideration p = 0.177; Involvement in decisions p = 0.166; One person with main responsibility p = 0.061*.

*OR, Odds ratio; CI, Confidence interval*.

## Discussion

This study provides new insight into the perceived healthcare needs, experiences and satisfaction among survivors of terrorism. Half of the survivors reported very high or high help needs for psychological reactions, and one in six reported very high or high help needs for attack-related physical health problems. The large proportion reporting high help needs suggests that a proactive outreach with screening assessments of all trauma survivors may be beneficial (Dyb et al., 2011). Participation in screening assessments might also have rendered the survivors more conscious about their help needs.

Most survivors scored the help they had received as equal to their needs. Yet one in five reported unmet needs for psychological reactions and one in seven for physical health problems. Unmet needs for psychological reactions were more common among survivors with more symptoms of posttraumatic stress and depression/anxiety both shortly and sometime after the attack. It seems plausible that there is a bidirectional relationship between increased symptom load and unmet needs; i.e., that higher morbidity increases the likelihood of unmet needs, and that insufficient treatment results in higher levels of symptoms. Unmet needs for psychological reactions were also associated with somatic symptoms and lower social support at wave 3, but not before. There is increasing awareness of the adverse physical health effects of chronic stress (Gupta, 2013). Furthermore, studies have shown that social support may reduce psychological stress, yet little is known about how persistent psychological stress may influence social relationships and support (Arnberg et al., 2012; Thoresen et al., 2014). Unfortunately, causal inferences cannot be drawn from the current analysis. The potential impact of untreated psychological stress on physical health and social relationships merits attention in future research.

With respect to physical health problems, unmet needs were associated with higher levels of posttraumatic stress, anxiety/depression and somatic symptoms, as well as lower social support at all survey waves. Interestingly, none of the injured survivors who were admitted to hospital after the attack reported unmet needs for physical health problems. It appears that the physical health of the severely injured was well cared for. In contrast, more than a third of the injured survivors who were not admitted to hospital and 12% of the survivors who were not injured reported unmet needs for attack-related physical health problems. In fact, the majority of survivors who reported very high/high help needs for attack-related physical health problems had not been physically injured. Our results underscore the importance of addressing the survivors' physical health in the aftermath of disasters, even in the absence of injuries. Previous studies have found somatic symptoms to be associated with higher levels of posttraumatic stress (Gupta, 2013) and the utilization of mental health services (Stene and Dyb, 2015) after disasters. Furthermore, somatic symptoms are associated with adverse psychosocial consequences and functional impairment (Campo et al., 1999).

Our findings suggest that the healthcare needs of survivors of immigrant origin require more attention. Compared to survivors of Norwegian origin, those with non-Norwegian origin were more likely to report unmet needs for attack-related physical health problems and to be less satisfied with the post-attack follow-up. It has been hypothesized that ethnic minorities tend to somatise distress but research results are inconsistent. Some studies have found no significant differences in somatization by ethnicity, (Bekker and Schepman, 2009) whereas others have indicated that somatization is either less (Bauer et al., 2012) or more (Aragona et al., 2005) common in ethnic minorities. Other possible explanations could be cultural or language-related barriers to satisfactory healthcare. The lower satisfaction with healthcare among immigrants agrees with findings from a Norwegian study in a non-disaster setting (Lien et al., 2008). Since participation rates generally tend to be lower among immigrants than non-immigrants, selection bias might occur. Indeed, in our study the loss to follow-up was greater among non-Norwegian survivors, and the results might not be representative for all non-Norwegian survivors. One should therefore be cautious when drawing conclusions concerning findings related to immigrant status.

This study applied univariable analyses to examine the relationship between survivor characteristics and perceived unmet healthcare needs. This information is valuable for identifying survivors at risk of having unmet help needs, improving the accessibility of healthcare and preventing unmet needs. Research in non-disaster settings indicates that perceived help needs are strongly associated with symptom severity (Mojtabai et al., 2002; Codony et al., 2009; Hargreaves et al., 2015). Identification of factors associated with unmet needs is therefore important in the planning of post-disaster outreach and the allocation of resources. Yet causal inferences cannot be drawn from the current analysis, and we cannot draw conclusions about why some survivors rated the help they had received as insufficient for their needs. The extent of self-perceived received help might reflect both quantity and quality of care. We lacked information about whether unmet needs were reported due to lack of healthcare or receipt of unsatisfactory healthcare. Furthermore, the amount of help offered to survivors with perceived unmet needs is unknown; it is possible that unidentified barriers prevented them from seeking or accepting care.

Although most survivors reported receiving help equal to their needs, a number of survivors reported their needs to be less than the help they received. This may be a natural consequence of the proactive outreach, as it comprised a screening assessment of all survivors irrespective of their symptom levels and functioning. Hence, survivors with no or few help needs may have received help that they believed to be unnecessary. This may represent a misallocation of resources, particularly for those who reported no or few needs but great or very great amounts of received help (Figure 1). On the other hand, studies in non-disaster settings have found that many individuals with mental disorders do not perceive their own need for help/treatment. It is possible that some of the survivors who reported receiving more help than they felt they needed might still have benefitted from it.

Most survivors reported both high satisfaction and benefit from the post-terror follow-up. Providing good healthcare experiences and satisfaction is increasingly becoming a priority for health authorities, and studies suggest that satisfaction is associated with better adherence to treatment. As previously mentioned, non-Norwegian survivors were less satisfied with the overall help they had received after the terrorist attack. The association remained significant after adjusting for other sociodemographic variables (Model 1, Table 3). A multivariable ordinal regression including perceived healthcare experiences indicated that the overall organization and accessibility of help services were particularly important quality measures in the aftermath of terrorism from the survivors' perspective (Model 2, Table 3).

## Strengths and limitations

This study included data from three waves of face-to-face interviews with young survivors of terrorism. One of the study strengths is the clear definition of the study population. The Utøya attack took place on a small island, where all survivors could be identified as having experienced a life-threatening event. In previous post-disaster studies the number and identity of directly affected survivors have often been unknown, and the respondents' trauma exposure may have diverged substantially. Furthermore, the first two waves of our study had an open cohort design, which allowed survivors to join the study at wave 2. This may have enhanced the response rate among survivors who were initially unable to participate due to poor health, thus providing a more representative sample than studies using closed cohorts (Stene and Dyb, 2016). All survivors were invited to participate, regardless of their receipt of healthcare services. Prior research on post-disaster health services has often included only recipients of specific services (Jackson et al., 2006). However, in the evaluation of outreach it is also important to assess those who did not receive care. Indeed, reaching out to everyone in need of follow-up is a considerable challenge after mass-casualty incidents.

A limitation of this study may be that we were unable to evaluate specific services individually. Respondents were asked to report their overall needs, received help and satisfaction with all the services they had received in relation to the terrorist attack over a period of more than 2.5 years. Therefore, we were unable to differentiate between their experiences with services in the early and the long-term phases after the attack. There may also have been recall bias due to the length of the period they were asked to evaluate. In this article, we assessed the survivors' subjective needs for, and receipt of, help and treatment. It is unknown to what extent appropriate, evidence-based care was provided to those in need. We also lacked information about the type of psychological reactions and physical health problems the survivors believed they needed help for. Moreover, there is no consensus on how unmet needs or satisfaction with healthcare should be measured. Satisfaction is a broad and subjective concept involving a variety of factors, including the survivors' expectations and preferences as well as the quality of the care they received (Bleich et al., 2009). We lacked information about the survivors' expectations and experiences with health services before the attack. Moreover, since few survivors rated low satisfaction with help services, we might have failed to identify predictors of low satisfaction (type 2 error). Furthermore, our results are based on responses from 52% of the survivors, and might not be representative of all survivors. There might be a particular risk of selection bias for results concerning immigrant status. A significantly higher number of survivors of non-Norwegian origin were lost to follow-up and were therefore not included in the current study. A Dutch study on survivors of a fireworks disaster found that health problems were associated with higher response among immigrant survivors; and with lower response among non-immigrants (Dijkema et al., 2005; Stene and Dyb, 2016).

External validity may be restricted by differences in the organization and financing of healthcare. This study was undertaken in a country with universal healthcare coverage, and participation in the proactive outreach program was free of charge. In countries with different organization of healthcare, access to health services may depend more on personal economy. Furthermore, our sample primarily comprised adolescents and young adults. The results are not necessarily representative of other age groups.

## Future research

More research is needed to understand the needs and experiences of terrorism survivors and their satisfaction with provided healthcare. A combination of quantitative and qualitative data could provide better insight into the reasons for, and nature of, satisfaction with services provided after terrorist events. Furthermore, subjective reports of healthcare needs, experiences and satisfaction should be combined with objective, accurate measures of type and frequency of provided care. In the evaluation of outreach, it is important to consider both clinical- and patient-centered outcomes and effective use of resources.

## Author contributions

LES contributed to the design, analyzed the data, and wrote the manuscript. TWL contributed to the statistical analyses and the drafting of the manuscript. GD was the study's project leader and contributed to drafting the manuscript.

### Conflict of interest statement

The authors declare that the research was conducted in the absence of any commercial or financial relationships that could be construed as a potential conflict of interest.
